# Merging pathology with biomechanics using CHIMERA (Closed-Head Impact Model of Engineered Rotational Acceleration): a novel, surgery-free model of traumatic brain injury

**DOI:** 10.1186/1750-1326-9-55

**Published:** 2014-12-01

**Authors:** Dhananjay R Namjoshi, Wai Hang Cheng, Kurt A McInnes, Kris M Martens, Michael Carr, Anna Wilkinson, Jianjia Fan, Jerome Robert, Arooj Hayat, Peter A Cripton, Cheryl L Wellington

**Affiliations:** Department of Pathology and Laboratory Medicine, The University of British Columbia, Vancouver, BC Canada; Graduate Program in Neuroscience, The University of British Columbia, Vancouver, BC Canada; Departments of Mechanical Engineering and Orthopaedics, The University of British Columbia, Vancouver, BC Canada; International Collaboration on Repair Discoveries, The University of British Columbia, Vancouver, BC Canada; Djavad Mowafaghian Centre for Brain Health, The University of British Columbia, Vancouver, BC Canada

**Keywords:** Traumatic brain injury, Animal model of traumatic brain injury, Animal model of closed head injury, Diffuse axonal injury, Microglia activation, Neuroinflammation, Tau hyperphosphorylation, Head kinematics, Head injury biomechanics, Impact-acceleration traumatic brain injury model, Surgery-free animal model of traumatic brain injury, Traumatic brain injury biomechanics

## Abstract

**Background:**

Traumatic brain injury (TBI) is a major health care concern that currently lacks any effective treatment. Despite promising outcomes from many preclinical studies, clinical evaluations have failed to identify effective pharmacological therapies, suggesting that the translational potential of preclinical models may require improvement. Rodents continue to be the most widely used species for preclinical TBI research. As most human TBIs result from impact to an intact skull, closed head injury (CHI) models are highly relevant, however, traditional CHI models suffer from extensive experimental variability that may be due to poor control over biomechanical inputs. Here we describe a novel CHI model called CHIMERA (Closed-Head Impact Model of Engineered Rotational Acceleration) that fully integrates biomechanical, behavioral, and neuropathological analyses. CHIMERA is distinct from existing neurotrauma model systems in that it uses a completely non-surgical procedure to precisely deliver impacts of prescribed dynamic characteristics to a closed skull while enabling kinematic analysis of unconstrained head movement. In this study, we characterized head kinematics as well as functional, neuropathological, and biochemical outcomes up to 14d following repeated TBI (rTBI) in adult C57BL/6 mice using CHIMERA.

**Results:**

Head kinematic analysis showed excellent repeatability over two closed head impacts separated at 24h. Injured mice showed significantly prolonged loss of righting reflex and displayed neurological, motor, and cognitive deficits along with anxiety-like behavior. Repeated TBI led to diffuse axonal injury with extensive microgliosis in white matter from 2-14d post-rTBI. Injured mouse brains also showed significantly increased levels of TNF-α and IL-1β and increased endogenous tau phosphorylation.

**Conclusions:**

Repeated TBI using CHIMERA mimics many of the functional and pathological characteristics of human TBI with a reliable biomechanical response of the head. This makes CHIMERA well suited to investigate the pathophysiology of TBI and for drug development programs.

**Electronic supplementary material:**

The online version of this article (doi:10.1186/1750-1326-9-55) contains supplementary material, which is available to authorized users.

## Background

Traumatic brain injury (TBI) is a leading worldwide cause of death and disability for persons under 45 years of age with a cost to society of over 17 billion USD per year. In the United States, the overall incidence of TBI is estimated to be 538 per 100,000 persons, which represents at least 1.7 million new cases per year since 2003 [1–3]. TBI incidence is reportedly lower in Europe (235 per 100,000) and Australia (322 per 100,000) [4, 5] although recent epidemiological data suggests far greater incidence (749 per 100,000) [6]. Mild TBI (mTBI), which includes concussion, comprises over 75% of all TBIs [3]. As mTBI is increasingly recognized as an injury for which medical attention should be sought, the reported incidence of mTBI is rising.

Falls are the most prevalent cause of TBI, and motor vehicle accidents and impacts against objects are also common causes [2, 4, 7]. TBI resulting from high-contact sports such as boxing, American football, ice hockey, soccer, and rugby account for almost 21% of all head injuries among children and adolescents, particularly for mTBI [8]. In these situations, the skull experiences an impact resulting in brain deformation and resulting injury that most often occurs without skull fracture. TBI is also considered a “signature injury” in modern warfare, as approximately 20% of veterans from the Iraq or Afghanistan wars are reported to have experienced a TBI, 80% of which involve both blunt impact and overpressure mechanisms [9–12]. Furthermore, the growing awareness that mTBI may have long-lasting and severe consequences [13–16] highlights the urgency to understand much more about the acute and long-term consequences of brain injury.

Rapid acceleration and rotation during impact TBI lead to vigorous movement and deformation of brain tissue within the skull that can result in contusions as the brain contacts the interior of the bony skull. These inertial and contact forces directly affect neurons, blood vessels, and glia, producing a primary injury that initiates secondary processes within hours to weeks after the initial injury [17–22]. These secondary changes lead to a plethora of events including edema, raised intracranial pressure, impaired cerebral blood flow, increased blood–brain barrier permeability, inflammation, axonal injury, calcium influx, elevated oxidative stress, free radical-mediated damage, excitatory neurotransmitter release, and cell death [17–22]. Although few treatment options are available for the primary injury, secondary injury pathways are potentially modifiable [23]. An increasingly wide variety of experimental animal models are therefore being developed to characterize secondary injury processes and for the evaluation of candidate therapeutic approaches.

No single animal model can replicate the entire spectrum of human TBI pathophysiology; therefore, several large and small animal models have been developed to mimic particular aspects. Popular rodent models include open-head injury models such us fluid percussion (FP) and controlled cortical impact (CCI) systems, and closed-head injury (CHI) models that use either gravity or mechanical methods to impact the intact skull (reviewed in [24]). Although FP and CCI models employ highly reproducible mechanical inputs and can mimic many pathological features of human TBI, the prominent tissue destruction and lack of head movement in these models decreases their resemblance to the majority of human injuries that occur due to impact and/or acceleration on an intact skull. In contrast, closed-head injury (CHI) models employ methods that do not generally cause overt brain tissue loss and can also allow rapid behavioral assessment of injury severity. As such, CHI models are considered by some to better mimic the majority of human TBI cases. However, a major limitation of most current CHI animal models is that the input parameters used to induce injury (e.g., mechanical loading, method of mechanical input, and response of the animal’s head to mechanical loading) are often poorly controlled, which can contribute to the considerable experimental variation across cognitive, histological, and biochemical outcome measures (reviewed in [24]).

Here we report a novel neurotrauma model called CHIMERA (Closed-Head Impact Model of Engineered Rotational Acceleration). CHIMERA was developed to address the absence of a simple and reliable model of rodent CHI that is representative of the majority of human TBI cases. CHIMERA is distinct from existing neurotrauma model systems in that it fully integrates biomechanical, behavioral, and neuropathological analyses after delivering impacts of defined energy to a closed skull with unconstrained head motion after impact. Here we show that repeated TBI (rTBI) in mice using CHIMERA reliably induces motor deficits, anxiety-like behavior, memory impairment, and leads to persistent diffuse axonal injury (DAI) with extensive white matter inflammation and increased phosphorylation of endogenous tau.

## Results

### Head kinematics during CHIMERA rTBI

**Figure 1:**
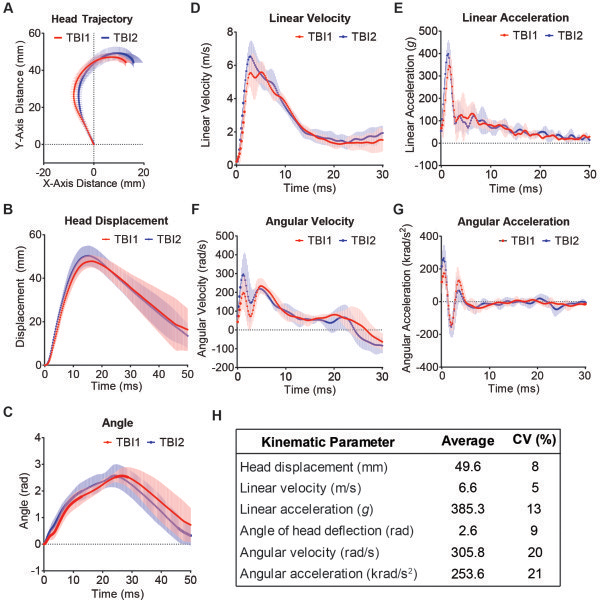
**Head kinematics during rTBI.** Head kinematic parameters during impacts were assessed in 8 mice subjected to rTBI. Data are represented as the means for each impact. **(A)** Head trajectory during the maximum acceleration phase in the sagittal plane following impact. **(B)** Head displacement-time graph following impact. **(C)** Head deflection is measured as the angle between the snout, side marker and the horizontal plane. Linear head velocity and linear head acceleration are depicted in **(D)** and **(E)**, respectively. **(F)** and **(G)** show angular head velocity and angular acceleration, respectively. Data in (A) are represented as mean ± 95% CI in both X- and Y- direction, respectively. Data in B-G are represented as mean ± 95% CI. **(H)** Summary of peak values of kinematic parameters averaged across all 8 rTBI mice. The coefficient of variation (CV) was calculated as the average of day 1 and day 2 peak values from all available recordings.

Analysis of high-speed videography (5,000 fps) was used to assess the biomechanical responses of the head in a group of 8 mice during CHIMERA rTBI at an impact energy of 0.5 J (Figure 1; peak kinematic parameters depicted in Figure 1H). Trajectories of the mouse head in the sagittal plane during peak acceleration following two impacts spaced at 24h are depicted in Figure 1A. Following vertical impact, the head followed a looped trajectory in the sagittal plane (Additional file 1: Figure S1, and Additional file 2: Movie S4). The average head trajectories following two repetitive TBIs in 8 mice were highly consistent (Figure 1A). The head traveled a peak linear displacement of 49.6 ± 3.5 mm (mean ± SD, same below) in 15.7 ± 2.4 ms (Figure 1B) and exhibited a peak angular deflection of 2.6 ± 0.28 rad in 24.8 ± 3.1 ms (Figure 1C). Peak linear velocity was 6.6 ± 0.8 m/s at 3.4 ± 1.0 ms (Figure 1D), and peak angular velocity was 305.8 ± 73.7 rad/s at 2.8 ± 1.9 ms following initial impactor contact (Figure 1F). The head experienced large linear and angular accelerations following impact, achieving peak linear acceleration of 385.3 ± 52 *g* at 1.5 ± 0.3 ms (Figure 1E), whereas the peak angular acceleration of 253.6 ± 69.0 krad/s^2^ was observed at 0.8 ± 1.1 ms (Figure 1G). As the head was stationary before impact, the change in head velocity (ΔV) equals peak head velocity and was found to be 6.6 m/s. The energy transferred from the piston to the head was 0.07 J.

Using the equal stress/equal velocity approach [25–27] to scale our murine kinematic data to human-equivalent values, ΔV was found to be comparable to National Football League (NFL) values and higher than Olympic boxing values, whereas scaled linear and angular velocity and acceleration parameters were lower than NFL values but comparable to Olympic boxing values (Additional file 3: Table S5).

### CHIMERA rTBI induces behavioral deficits

**Figure 2:**
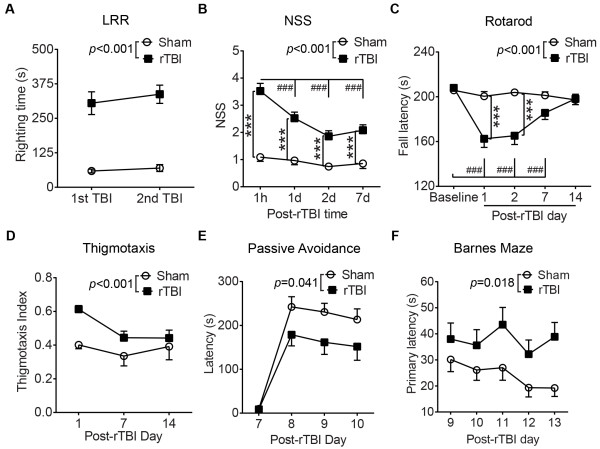
**CHIMERA rTBI induces behavioral deficits. (A)** Duration of loss of righting reflex (LRR) was assessed immediately following the sham or TBI procedure. Cohort size: Sham, N = 31; rTBI, N = 39. **(B)** Neurological severity score (NSS) was assessed at 1h, 1d, 2d, and 7d post-rTBI. Cohort size: Sham (1 h: N = 34, 1d: N = 31, 2d: N = 35, 7d: N = 21); rTBI (1 h, 1d and 2d: N = 42, 7d: N = 25). **(C)** Motor performance was assessed on an accelerating rotarod at 1d, 2d, 7d and 14d post-rTBI. Cohort size: Sham (1d and 2d: N = 35, 7d: N = 21, 14d: N = 15); rTBI (1d and 2d: N = 41, 7d: N = 25, 14d: N = 15). **(D)** Thigmotaxis was quantified at 1d, 7d and 14d post-rTBI. Cohort size: Sham (1d: N = 23, 7d: N = 16, 14d: N = 15); rTBI (1d: N = 24, 7d: N = 10, 14d: N = 15). Data in A-D were analyzed by repeated measures two-way ANOVA followed by the Holm-Sidak post-hoc test. **(E)** Working memory was assessed by the passive avoidance test from 7d to 10d post-rTBI.. **(F)** Spatial reference memory was assessed by Barnes maze from 9d to 13d post-rTBI. Data at each time point represent the mean of four trials. Data in E and F (Cohort size: N = 15/group) were analyzed by repeated measures two-way ANOVA. For all graphs, data are presented as mean ± SEM. For all graphs, * indicates a significant rTBI effect within a particular time point and # indicates a significant time effect within each group. ***: *p* < 0.001. ###: *p* < 0.001.

Loss of righting reflex (LRR) in animals after TBI is considered analogous to loss of consciousness in humans after TBI and can be considered as a behavioral indicator of injury severity [28]. Mice subjected to CHIMERA rTBI showed significantly increased LRR duration compared to sham animals (Figure 2A; TBI effect: *F*(1, 65) = 59.61, *p* < 0.001). LRR duration was consistent between the first and second impacts (Figure 2A). We further assessed injury severity using the Neurological Severity Score (NSS), which is a composite of ten tasks that assess motor reflexes, alertness, and physiological behavior [29] (Additional file 4: Table S6). The NSS of injured animals was significantly higher than sham mice from 1h to 7d post-procedure (Figure 2B, TBI effect: *F*(1, 191) = 44.12, *p <* 0.001). In injured mice, the NSS score showed maximum deficits at 1h post-procedure (*p* < 0.001) followed by steady spontaneous improvement over 1-7d, albeit remaining significantly higher than sham animals at each post-rTBI time point (Figure 2B, *p* < 0.001). Similarly, rTBI significantly impaired motor performance from 1-7d post-injury as indicated by reduced fall latencies on an accelerating rotarod compared to sham controls (Figure 2C, TBI effect: *F*(1, 220) = 11.99, *p* < 0.001). Fall latencies showed both time effects (*F*(4, 220) = 13.70, *p* < 0.001) and TBI × Time interaction (*F*(4, 220) = 11.22, *p* < 0.001). Motor deficits in injured mice peaked at 1d (*p* < 0.001) and returned to baseline conditions by 14d post-injury (*p* = 0.22), whereas sham mice did not show any motor deficit (*p* > 0.82). Injured mice showed anxiety-like behavior as indicated by significantly increased thigmotaxis in an open field test (Figure 2D, TBI effect: *F*(1, 53) = 12.30, *p* < 0.001). The thigmotactic behavior of both groups significantly declined over time in a similar trend (Figure 2D, Time effect: *F*(2, 53) = 5.45, *p* = 0.007; TBI × Time interaction insignificant). Open field thigmotaxis was not affected by gross motor activity as no significant differences in total distance traveled or time immobile were observed between injured and sham-operated mice (Additional file 5: Figure S2). Repeated TBI also induced working memory impairment as indicated by decreased latencies to enter the darkened compartment on the passive avoidance task (Figure 2E, TBI effect: *F*(1, 28) = 4.6, *p* = 0.041). For all mice, a main effect of Day indicated that mouse behavior changed over the training and testing sessions evaluated in this study (*F*(3, 84) = 58.55, *p <* 0.001). For the main effect of Day, pairwise comparisons indicated that mice entered the darkened compartment significantly faster on Day 7 (*p* < 0.001) than on Days 8–10 (*p* > 0.05), signifying that all mice remembered the shock to some degree (Figure 2E). Injured mice also showed spatial reference memory impairment as indicated by increased latencies to locate the escape hole on the Barnes maze (Figure 2F, TBI effect: *F*(1, 28) = 6.27, *p* = 0.018). Under our experimental conditions, cognitive performance did not spontaneously resolve by the end of our testing period.

### CHIMERA rTBI induces widespread persistent diffuse axonal injury

**Figure 3:**
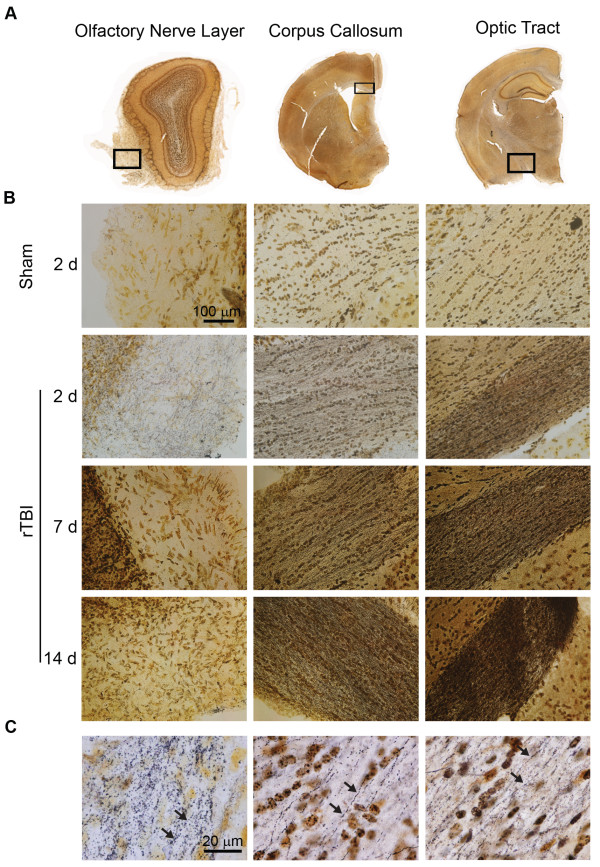
**CHIMERA rTBI induces diffuse axonal injury.** Axonal degeneration was assessed by silver staining at 2, 7, and 14d post-rTBI. **(A)** Coronal sections showing white matter areas including the olfactory nerve layer, corpus callosum, and optic tract with regions of prominent silver staining indicated by black rectangles. **(B)** Representative 40X-magnified images of the same brain regions in sham-operated (upper row) or rTBI-induced (lower three rows) animals at the indicated time points. **(C)** 100X-magnified images of the same brain regions in rTBI-induced animals. Axonal varicosities are indicated by arrows.

**Figure 4:**
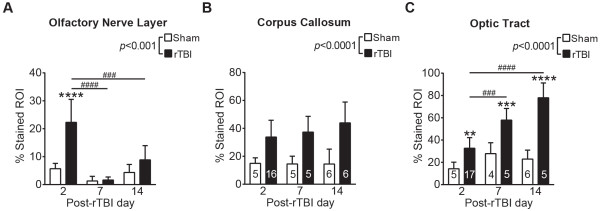
**Quantitative analysis of silver stain images.** Silver stained images were quantified by calculating the % of region of interest (ROI) in the white matter tract area that was stained with silver. Bars indicate mean ± SD percent of ROI showing positive signal in sham and rTBI-induced animals in **(A)** olfactory nerve layer, **(B)** corpus callosum and **(C)** optic tract. Data were analyzed using two-way ANOVA followed by a Tukey post-hoc test. Cohort size: olfactory nerve layer: Sham (2d: N = 4, 7d: N = 5, 14d: N = 6); rTBI (2d: N = 8, 7d and 14 d: N = 5); corpus callosum: Sham (2d and 7d: N = 5, 14d: N = 6); rTBI (2d: N = 16, 7d: N = 5, and 14 d: N = 6); optic tract: Sham (2d: N = 5, and 7d: N = 4, 14d: N = 6); rTBI (2d: N = 17, 7d and 14d: N = 5). For all graphs, * indicates a significant rTBI effect within a particular time point and # indicates a significant time effect within rTBI group. **: *p* < 0.01, ***: *p* < 0.001, ****: *p* < 0.0001. ###: *p* < 0.001, ####: *p* < 0.0001.

Silver staining was used to assess post-rTBI axonal damage at 2, 7, and 14d post-rTBI (Figures 3 and 4). rTBI brains revealed widespread axonal injury, as indicated by intense punctate and fiber-associated argyrophilic structures in several white matter tracts including the olfactory nerve layer of the olfactory bulb, corpus callosum, and optic tracts (Figure 3A and B, Figure 4). Axonal injury was observed at both coup (corpus callosum) and contrecoup (optic tract) regions, indicating a diffuse injury pattern. High-magnification of the affected areas at 100X revealed numerous axonal varicosities (Figure 3C, arrows), which is a characteristic histological feature of human axonal pathology after TBI [30]. Quantitative analysis revealed significant silver uptake in the injured olfactory nerve layer (TBI effect: *F*(1, 27) = 16.89, *p* < 0.0001), which was maximum at 2d (*p* < 0.001) and returned to sham levels over 7-14d (Figure 4A). On the other hand, rTBI induced persistent silver stain uptake in the corpus callosum (Figure 4B, TBI effect: *F*(1, 37) = 41.54, *p* < 0.0001; Time effect insignificant) (Figure 4C). In the optic tract, silver uptake was most intense (Figures 3B and 4C TBI effect: *F*(1, 36) = 107.4) and there was a significant time and injury interaction (TBI x Time interaction: *F*(2, 36) = 11.66), indicating persistent increase in axonal degeneration in contrecoup regions.

### CHIMERA rTBI induces widespread microgliosis and increases proinflammatory cytokine levels

**Figure 5:**
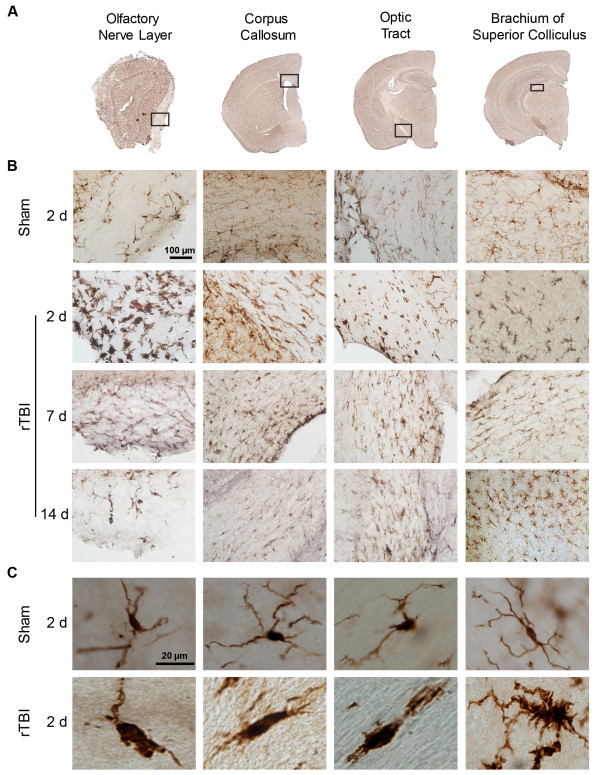
**CHIMERA rTBI induces widespread microglial activation.** Microglial activation was assessed using Iba-1 immunohistochemistry at 2, 7, and 14d post-rTBI. **(A)** Representative images of Iba-1 stained coronal sections of olfactory bulb and brain. Areas with prominent microglial activation are indicated by black rectangles. **(B)** Representative 40X-magnified images of the same white matter tract regions showing resting microglia in sham brains (upper row) and activated microglia in injured brains (lower three rows) at the indicated time points. **(C)** Representative 100X-magnified images showing the morphology of Iba-1-stained resting microglia in sham (upper row) and activated microglia in rTBI (lower row) brains.

**Figure 6:**
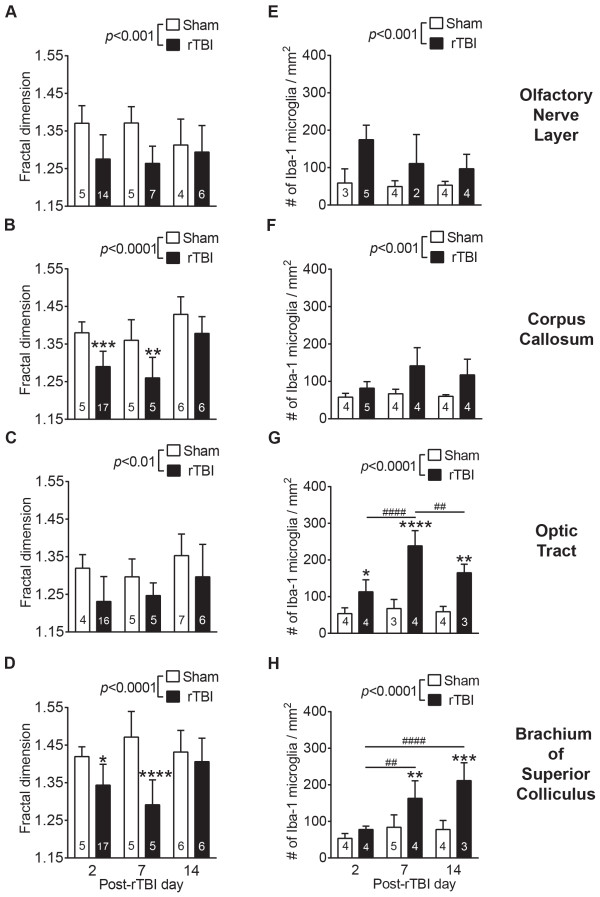
**Quantitative analysis of microglial response to rTBI.** Bar graphs in the left column **(A-D)** indicate mean ± SD fractal dimension for microglial morphology in **(A)** olfactory nerve layer, **(B)** corpus callosum, **(C)** brachium of superior colliculus, and **(D)** optic tract. Bar graphs in the right column **(E-H)** show mean ± SD number of Iba-1 positive cells per mm^2^ in the same white matter regions. Data were analyzed by two-way ANOVA followed by a Tukey post-hoc test. Numbers inside the bars indicate sample size. For all graphs, * indicates a significant rTBI effect within a particular time point while # indicates a significant time effect within rTBI group. *: *p* < 0.05, **: *p* < 0.01, ***: *p* < 0.001, ****: *p* < 0.0001. ##: *p* < 0.01, ####: *p* < 0.0001.

Using Iba-1 immunohistochemistry, we observed significantly increased microglial activation throughout several white matter tracts including the olfactory nerve layer, corpus callosum, optic tracts, and brachium of superior colliculus of injured brains compared to the sham controls as assessed using both fractal analysis and microglial density (Figures 5 and 6). Quantification of microglial morphology by fractal analysis revealed that microglia in sham animals displayed highly ramified and extensively branched processes that are characteristic of the resting state (Figure 5C, upper row, Figure 6A-D, open bars). By contrast, microglia in the corpus callosum, brachium of superior colliculus, and olfactory nerve layer of injured animals had predominantly hypertrophic to bushy morphology with primary branches only, whereas those in the optic tract showed amoeboid morphology characteristic of highly activated microglia (Figure 5C, lower row, Figure 6A-D, black bars). Quantitative analysis showed significant and persistent microglial activation in the injured olfactory nerve layer (TBI effect: *F*(1, 35) = 13.64, *p* = 0.0008), optic tract (TBI effect: *F*(1, 37) = 9.77, *p* = 0.0034), corpus callosum (TBI effect: *F*(1, 38) = 29.51, *p* < 0.0001), and brachium of superior colliculus (TBI effect: *F*(1, 38) = 24.5, *p* < 0.0001) as soon as 2d (Figure 6A, B, C and D).

In addition to changes in microglial morphology, we observed significant increases in the number of microglia in the same white matter regions including the olfactory nerve layer (Figure 6E, TBI effect: *F*(1, 16) = 21.53, *p* = 0.0003), optic tract (Figure 6G, TBI effect: *F*(1, 16) = 90.30, *p* = 0.0001), corpus callosum (Figure 6F, TBI effect: *F*(1, 19) = 22.25, *p* = 0.0002) and brachium of superior colliculus (Figure 6H, TBI effect: *F*(1, 18) = 34.85, *p* < 0.0001), indicating that injury induced proliferation or recruitment of immune cells. In olfactory bulb (Time effect insignificant), corpus callosum (Time effect insignificant) and optic tract, microglia cell number was persistently increased from 2d up to 14d (*p* < 0.05). In the brachium of superior colliculus, a delayed but persistent increase in the Iba-1 positive cell number was observed from 7d to 14d (*p* < 0.01).

**Figure 7:**
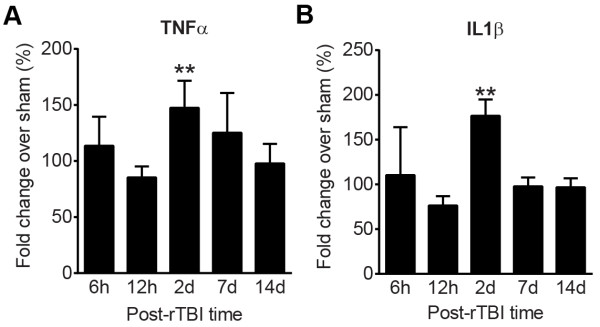
**CHIMERA rTBI increases proinflammatory cytokine levels.** Bar graphs represent mean ± SD % fold change in TNFα **(A)** and IL-1β **(B)** levels in rTBI brain lysates compared to the levels in the sham brain lysates at the respective time points. For both graphs, ** indicates *p* < 0.01 in comparison of rTBI vs sham, using multiple t-tests with Bonferroni corrections for multiple comparisons (*p* = 0.05/5 = 0.01).

In addition to the microglial response, we also measured protein levels of the proinflammatory cytokines TNFα and IL-1β in half brain homogenates. Protein levels of TNFα (Figure 7A) and IL-1β (Figure 7B) were significantly higher at 2d post-TBI compared to the respective sham levels (*p* < 0.01).

### CHIMERA rTBI increases endogenous tau phosphorylation

**Figure 8:**
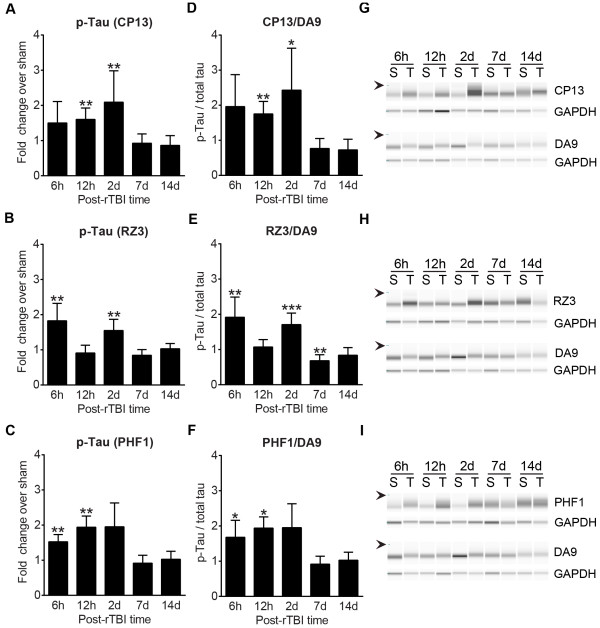
**CHIMERA rTBI increases endogenous tau phosphorylation.** Tau phosphorylation was analyzed using the Simple Western system (ProteinSimple). The graphs in the left column **(A-C)** depict fold change in endogenous phosphorylated tau levels in rTBI half-brain homogenates compared to the sham brains using antibodies CP13 (pSer202 and pThr205, Panel **A**), RZ3 (pThr231, Panel **B**) and PHF1 (pSer396 and pSer404, Panel **C**), respectively. Graphs in the middle column **(D-F)** depict quantitation of phosphorylated tau as a proportion of total tau (DA9). Representative digital immunoblots of phosphorylated and corresponding total tau are depicted in the right column **(G-I)**. Arrows on the left of the blots indicate molecular weight marker at 66 kDa. Data are presented as the mean ± SD fold change in rTBI compared to the respective shams at each time point. For all graphs, *, ** and *** indicate *p* < 0.01 for the comparison of rTBI vs respective sham values, using multiple t-tests with Bonferroni correction for multiple comparison (*p* = 0.05/5 = 0.01).

We next assessed the phosphorylation levels of endogenous murine tau using three antibodies directed against different phosphorylation sites, namely CP13 (pSer202), RZ3 (pThr231), and PHF1 (pSer396 and pSer404). Total murine tau levels were determined by the antibody DA9. Simple Western analysis showed significantly increased phosphorylation of all the probed epitopes in rTBI brain lysates at 6h, 12h, and 2d compared to the respective sham brain lysates (Figure 8A-C and 8G-I, *p* < 0.01). The change in tau phosphorylation reflected a significant increase in the ratio of phosphorylated tau:total tau, but not a change in total tau levels (Figure 8D-F and 8G-I, *p* < 0.01).

## Discussion

**Figure 9:**
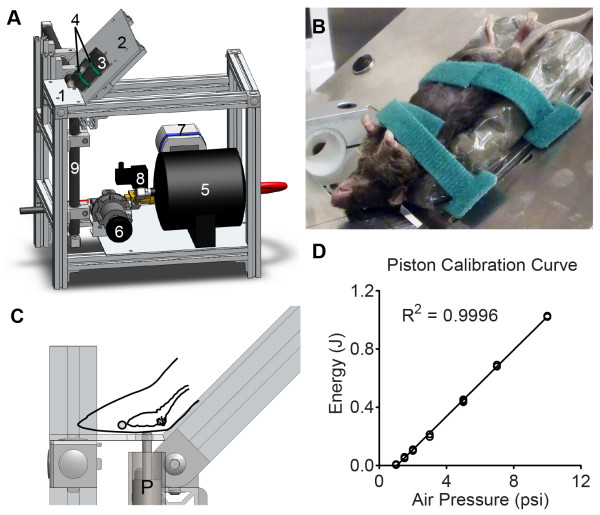
**CHIMERA device and mouse head positioning. (A)** The picture depicts the CHIMERA device. Various parts are labeled with numbers as follows: 1. head plate, 2. body plate, 3. animal bed, 4. Velcro straps, 5. air tank, 6. air pressure regulator, 7. digital pressure gauge, 8. two-way solenoid valve, 9 vertical piston barrel. **(B)** Close-up view of animal strapped on the holding platform. **(C)** Location of impact relative to the mouse head and brain. P: impact piston. **(D)** Air pressure-energy calibration curve was obtained by driving a 50 g piston at increasing air pressure values and calculating the resultant impact energy. The graph depicts three measurements for each air pressure value.

The major goal of this study was to develop a simple, reliable model of murine CHI that replicates fundamental aspects of human impact TBI through precise delivery of known biomechanical inputs. CHIMERA fulfills these criteria and offers several key advantages over existing rodent TBI models. CHIMERA is completely nonsurgical and requires only isoflurane anesthesia, therefore enabling immediate neurological severity assessments using LRR and NSS measures. Being nonsurgical, CHIMERA is ideal for studies investigating multiple impacts as well as the long-term consequences of impact TBI. These advantages overcome many limitations of surgically-induced TBI models, including longer exposure to multiple anesthetic agents, such as opioid analgesics (buprenorphine) and sedatives (xylazine) that can interfere with rapid neurological assessment. Surgical models are also low throughput, require extensive operator training, and have limited suitability for studies involving repetitive TBI or long-term TBI outcomes. By contrast, CHIMERA produces injury using a simple, reliable, and semi-automated procedure that requires <10 min per animal to produce defined injury (Figure 9). As the biomechanical input parameters are highly adjustable across impact energy, velocity, and directional parameters, CHIMERA offers a wide dynamic range of precisely controllable inputs to reproduce specific conditions that occur in human impact TBI. Importantly, the kinematic analyses facilitated by CHIMERA enable head motion parameters to be integrated with behavioral and neuropathological outcomes, potentially enabling greatly improved translational relevance to human TBI. CHIMERA produces diffuse injury characterized by activation of inflammatory reactions, axonal damage, and tau phosphorylation, replicating many aspects of the neuropathology of human impact TBI without overt focal damage. Taken together, these attributes make CHIMERA a valuable new model to investigate the mechanisms of TBI and for use in preclinical drug discovery and development programs.

In this study, a 50 g piston was used to deliver an input impact with a kinetic input energy of 0.5 J. Calibration curves show that the 50 g piston has an energy range of 0.01 J to 14.0 J (useful range for murine TBI is 0.1 J to 1 J) in minimum steps of 0.01 J, with highly reproducible performance (Figure 9D). Adjusting the piston mass and dimensions will allow other biomechanical parameters to be controlled, so that it may be possible to experimentally model the biomechanical conditions observed under different types of human impact TBI.

High-speed video integrated into CHIMERA enables measurement of mouse head kinematic parameters that can be scaled to humans. The most-commonly used method for scaling kinematic parameters between humans and animals is based on the equal stress/equal velocity approach [25–27]. Thus, velocity does not scale and is the same for human and animal data. We used a scaling factor λ [λ = (mass of human brain/mass of mouse brain)^1/3^ = 13.8] to estimate the human head-equivalent kinematic parameters from our animal data. Although this scaling approach has been widely used in the study of impact biomechanics [25–27], and has been applied to a rat CHI model [25], it is important to be cautious in its extrapolation as the human and rodent brains differ in geometry, white:grey matter ratio, ventricular volume and position, and cortical folding. All of these factors render the “scaling” of rodent data to human to be an approximation at best. Nevertheless, under our experimental conditions, behavioral and neuropathological changes reliably occurred at lower scaled values of all kinematic parameters (except for impact duration) than those reported for NFL concussions [31, 32]. Because clinical mTBI can occur under many different circumstances (e.g., falls, passengers and pedestrians in motor vehicle accidents, non-NFL sports), an important area for future research will be to determine how various impact characteristics lead to functional and biochemical changes. The precision and flexibility CHIMERA offers with respect to impact parameters will help to refine the relationship between impact characteristics and physiological outcomes.

An additional advantage of CHIMERA is that it overcomes much of the variability observed with most CHI models. Typical weight-drop models [29, 33] have poor control over biomechanical input parameters, including friction and air resistance inside the guide tube that may contribute to variable outcomes. High incidence of skull fractures and limited dynamic range pose additional challenges in comparing results using weight-drop TBI models across different laboratories [24]. Head movement during many CHI impact models is often at least partially restricted by anchoring the head within a stereotaxic frame or by resting the animal on various types of support. In a recent modification, Kane et al. supported mice on a piece of aluminum foil that ruptures upon impact and leads to a 180° rotation of the animal [34]. While this modification allows unrestricted head movement, it still includes possible sources of variability including stretching of the aluminum foil before yielding to the force generated by the weight-drop and less reliable and adjustable positioning of impact location compared to CHIMERA.

Loss of consciousness for <30 min is one of the clinical criteria for mTBI [35] and the analogous measure in mice is LRR. In animal TBI models, an LRR of 15–30 min is considered moderate-severe TBI while an LRR of <15 min is considered mild TBI [28]. The average LRR duration after CHIMERA-rTBI was 5.3 min, indicating mild TBI. Interestingly, a second TBI occurring 24 h after the first impact did not prolong LRR time. Post-concussion patients may also show balance difficulties or postural instability up to several days [36–38], as well as mood changes such as irritability or anxiety [38, 39]. Though general locomotor activities were not severely affected (Additional file 5: Figure S2), CHIMERA-rTBI resulted in deficits in fine motor coordination and neurological performance. Similar changes have also been reported by other groups [40, 41]. CHIMERA-rTBI also increased thigmotaxis in TBI animals, suggesting an anxiety-like behavior [42]. Our model also revealed deficits in both working and spatial reference memory as assessed by the passive avoidance test and Barnes maze, respectively. Intriguingly, recovery of motor performance was faster than cognitive performance under the conditions of our study.

Diffuse axonal injury (DAI) is one of the characteristic pathologies of TBI [30, 43]. Using silver staining, we observed increased argyrophilic fibers and punctate structures in several white matter tracts across the brain, suggesting a diffuse pattern of damaged axons. Axonal varicosities, a classical feature of DAI in humans, were also present [30]. Interestingly, both white matter areas that were close to (e.g., corpus callosum) and distant from (e.g., optic tract and olfactory nerve layer) the impact site were affected, suggesting that both coup and contrecoup injuries are present in our model. Affected white matter areas, including the corpus callosum, optic tract, and the olfactory system, have been reported in other CHI models that induce impact at the superior side of skull [44–47]. Several white matter areas showed increased argyrophilic staining as well as reactive microgliosis following rTBI, suggesting a possible relationship between axonal damage and neuroinflammation, in agreement with previous reports [46, 48, 49]. Interestingly, axonal injury in the optic tract continued to increase from 2 to 14d suggesting that ongoing secondary injury processes overwhelmed endogenous repair mechanisms in the time frame examined in this study. Moreover, optic tract, a contrecoup injury site, showed the most intense silver uptake, which is in agreement with the common clinical observation that contrecoup injuries are more severe than the coup injuries [50]. On the other hand, axonal injury was resolved in the olfactory neuronal layer within 7d, suggesting efficient neural repair or active neuroregeneration in this region. Future studies will be designed to assess changes in cognitive functions and the dynamics of axonal injury across several brain regions in our model over longer-term (up to 6 months) post-rTBI follow up.

Human and experimental TBI induce rapid neuroinflammatory responses as demonstrated by changes in cytokine levels (e.g., IL-1β and IL-6) and microglial activation [44, 51–55]. Under our injury conditions, rTBI led to elevated IL-1β and TNF-α levels at 2d after injury, which was accompanied by histological evidence of microgliosis. Because our animal ethics committee required the use of meloxicam for pre-emptive pain control, it is possible that an inflammatory response occurring during the first few hours after impact [52, 54, 55] was suppressed [56]. Iba-1-positive activated microglia were particularly evident along white matter tracts throughout the brain, whereas grey matter was essentially spared. Microglial activation as assessed by fractal analysis and cell density was significant at 2d and persistent until 14d. However, as Iba-1 does not distinguish the source of immune cells, the increase in cell number in this study may be due to proliferation of resident immune cells in the brain or recruitment of immune cells from the periphery, or both.

Hyperphosphorylation of the cytoskeletal protein tau is a pathological event observed in many neurodegenerative diseases including Alzheimer’s disease [48] and chronic traumatic encephalopathy [49]. In our model, we demonstrate that endogenous tau hyperphosphorylation is an early and dynamic event after rTBI in wild-type mice, again, in agreement with other models of CHI [57, 58]. It should, however, be noted that post-TBI changes in phosphorylation of murine tau does not predict whether human tau will show similar dynamics. Further experiments using transgenic human tau mice will be required to investigate the influence of rTBI on tau deposition.

## Conclusions

Here we report a novel, surgery-free CHI model that fully integrates biomechanical, functional, and neuropathological characteristics of TBI. CHIMERA allows precise control over mechanical inputs allowing reproducible head kinematics. Our study also shows that CHIMERA-TBI reliably replicates several key behavioral, biochemical, and neuropathological characteristics of human TBI including axonal injury, neuroinflammation, and functional deficits. Future studies will be conducted to characterize, in more detail, the relationships between kinematics and the resulting behavioral and neuropathological responses across a variety of impact parameters. The significant advantages CHIMERA offers over comparable rodent TBI models are expected to facilitate the acquisition of preclinical data with improved relevance to human TBI, thereby accelerating the pace of successful research to understand the mechanisms of TBI and to develop effective therapeutic approaches for this devastating condition.

## Methods

### CHIMERA impactor

The CHIMERA impactor consists of an aluminum frame that supports an animal holding platform above a pneumatic impactor system (Figure 9). The animal holding platform is composed of a fixed head plate that supports the animal’s head in a supine position and a body plate that positions and secures the animal’s torso. The head plate has a hole through which the tip of the impactor piston is projected to contact the animal’s head. A cushion made from closed-cell foam surrounds the hole to minimize rebound impact when the animal’s head falls back upon the head plate. Two perpendicular lines across the piston hole act as crosshairs for aligning the animal’s head over the hole. The body plate holds a restraint system consisting of an animal bed of closed-cell foam contoured to the shape of the animal’s body and two Velcro straps. The animal holding platform is attached to the frame by hinges and its angle of inclination can be adjusted. In this study, the angle was set to approximately 32° such that the frontal and parietal bones lie flat over the hole in the head plate, thus delivering impact to the dorsal cortical region.

The pneumatic impactor system includes an accumulator air tank, pressure regulator, digital pressure gauge, two-way solenoid valve, and trigger button. The pressure regulator and digital pressure gauge allow precise adjustment of air pressure to 0.1 psi (0.69 kPa), enabling accurate delivery of piston velocity and impact energy. Impact is induced with a 50 g free-floating chrome-coated steel piston whose trajectory is constrained to linear motion by a steel barrel. The piston barrel has an array of holes drilled near the muzzle end to vent air and equalize the pressure as the piston moves past them towards the impact site. The piston is accelerated by a controlled pulse of compressed air along the length of the barrel until it clears the venting holes.

The CHIMERA impactor was calibrated by measuring the exit velocity of the piston at various air pressures (0.5, 1, 1.5, 2, 3, 5, 7, and 10 psi) to determine the relationship between air pressure and piston velocity. Three measurements were taken at each pressure value. Each impact event was recorded by a high-speed video camera at 10,000 fps and tracked by video motion analysis software (TEMA Motion, Image Systems AB, Sweden). A 2nd order polynomial curve was used to fit the data. The r^2^ value was 0.9996 (Figure 9D). Using this curve, the desired impact velocity or energy can be independently interpolated. By choosing the appropriate air pressure, impacts of input energy ranging from 0.01 J to 1 J can be precisely generated.

### CHIMERA TBI procedure

All animal procedures were approved by the University of British Columbia Committee on Animal Care (protocol # A11-0225) and were carried in strict accordance with the Canadian Council on Animal Care guidelines. Male C57Bl/6 mice (mean ± SD body weight 33.9 ± 4.6 g) at 4 months of age were housed with a reversed 12h light-12h dark cycle for at least 10 days before TBI. Animals were anaesthetized with isoflurane (induction: 4.5%, maintenance: 2.5-3%) in oxygen (0.9 L/min). Lubricating eye ointment was applied to prevent corneal drying. Meloxicam (1 mg/kg) and saline (1 mL/100 g body weight) were administered by subcutaneous injections for pain control and hydration, respectively. Animals were placed supine in the holding bed such that the top of the animal’s head lay flat over a hole in the head plate, aligned using crosshairs such that the piston strikes the vertex of the head covering a 5 mm area surrounding the bregma (Figure 9B and C). Impact was induced by pressing a trigger button that simultaneously fires the piston and when connected, activates a high-speed camera to record the resulting head trajectory. Isoflurane delivery was immediately stopped and the animal was continuously monitored until fully ambulatory. Total duration of isoflurane exposure was ~ 4–8 min. Twenty-four hours after the first impact, a second identical impact was delivered. Sham animals underwent all of these procedures, except for the impact. Approximately 3% of animals did not regain consciousness for > 45 min or displayed severe motor dysfunction after TBI, and were thus euthanized.

### High-speed videography and kinematic analysis

For kinematic analysis, an independent cohort of 8 mice was subjected to rTBI and impact events were recorded at 5,000 frames per second using a high-speed video camera (Q-PRI, AOS Technologies, Switzerland). Head motion was tracked using two markers, one being non-toxic paint applied on lateral side of head to mark the cheek area (Additional file 1: Figure S1, yellow arrow in the first image). Because the skin is loose over the bony skull, we also marked the position of the maxilla by wrapping dental floss positioned just caudal to the upper incisors around the animal’s snout (Additional file 1: Figure S1, red arrow in the first image). Videos were analyzed using ProAnalyst motion analysis software (v 1.5.6.8, Xcitex Inc., Woburn, MA). The X and Y coordinates of the position of each marker were tracked on a frame-by-frame basis and were processed with a 400-Hz low-pass Butterworth filter to mitigate the noise in the recorded measurements. Velocities and accelerations were determined by discrete differentiation of the position data. Resultant linear velocity and acceleration were calculated as the magnitude of their respective X and Y components. Linear kinematic parameters were assessed from trajectories obtained by tracking the paint mark. Angular rotation of the head during TBI was determined by the angle of the line joining the dental floss and the paint mark with the horizon. After video capture, the dental floss was removed. Energy transferred from the piston to the head was determined using the equation KE = 0.5 × M_e_ × ΔV^2^, where M_e_ is the effective mass and is approximated by head mass (3.4 g) and ΔV is a change in head velocity. A scaling factor λ [λ = (mass of human brain/mass of mouse brain)^1/3^ = 13.8] was used to estimate the human head-equivalent kinematic parameters from the animal data.

### Behavioral analyses

LRR was calculated as the time interval from isoflurane discontinuation to the first sign of righting after each impact. Neurological impairment was assessed using the NSS [29] determined at 1h and at 1, 2, and 7d following the second TBI. NSS is a composite of ten different tasks that assess motor function, alertness, and physiological behavior (Additional file 4: Table S6). One point is awarded for the lack of a tested reflex or for the inability to perform the tasks, and no point for succeeding the task. A maximal NSS of 10 points thus indicates severe neurological dysfunction, with failure of all tasks. Motor performance was evaluated at 1, 2, 7, and 14d after the second TBI using an accelerating rotarod as previously described [46]. Open field activity was assessed at 1, 7, and 14d after the second TBI using a Plexiglas box (14” × 24” × 14”). The floor of the box was virtually divided into 60 equal squares using an overhead digital camera and video tracking software (ANY-maze, v. 4.99, Stoelting Co, Wood Dale, IL). The field was further subdivided into a peripheral zone along the walls of the open field consisting of 28 squares that surrounded a central zone consisting of 32 squares. The animal was placed in the center of the box and spontaneous activity was recorded for 10 min, including quantification of the total distance traveled and immobile time. The thigmotaxis index (TI) was calculated as: TI = (T_P_-T_C_)/(T_P_ + T_C_) where T_P_ and T_C_ represent the time spent in the peripheral and central zones, respectively. Working and spatial reference memories were assessed from 7d to 13d after the second TBI using the passive avoidance task (7-10d) and the Barnes maze (8-13d), respectively. Passive avoidance testing was conducted in a device that consisted of two adjoining compartments, one illuminated (20.3 × 15.9 × 21.3 cm) and one darkened (20.3 × 15.9 × 21.3 cm), divided by a guillotine-style door (Med Associates Inc., St. Albans, VT). The floor of the compartments consisted of steel rods capable of delivering an electric foot-shock. The electric shock was delivered by a programmable animal shocker (Med Associates Inc.). Each session consisted of placing mice into the illuminated compartment and using a timer to record the latency of the mice to cross into the darkened compartment. On 7d after the second TBI (training), mice received an electric foot-shock (0.3 mA, 2 s) as soon as they crossed from the illuminated into the darkened compartment. Following foot-shock, mice were removed from the apparatus and returned to their home cage. On 8d to 10d after the second TBI mice were tested for memory retention. The latency for the mice to cross into the darkened compartment was recorded. No shock was delivered during testing. Mice that did not cross over into the darkened compartment were allowed to remain in the illuminated compartment for the full 5 min and assigned a latency of 300 s. Barnes maze testing was conducted on a grey circular platform (91 cm diameter) with 20 circular holes (5 cm diameter; Stoelting Co) located in a 3.0 × 3.6 m room. An escape box was positioned beneath one of the holes. Extra-maze visual cues consisted of black and white images (cross, lightning bolt, smiley face) printed on glossy white paper and placed on the walls surrounding the maze. Other visual cues included a cart with a laptop and the experimenter. A digital camera was placed 4 m above the center of the maze to record trials for the ANY-maze tracking system. For motivational purposes, mice were food restricted to 1 g of food per day from 8-13d after the second TBI and maintained at 90% of free feeding body weight. During testing, mice were exposed to aversive stimuli in the form of two lights (100 W) positioned at either side of the maze and a buzzer (2.9 kHz, 65 dB) that hung 10 cm above the maze. On 8d after the second TBI, mice completed one 5-min habituation trial to become familiar with the maze environment and to practice descending into the escape box. On 9d to 13d after the second TBI, mice were tested for memory retention. Mice were given four trials per day (15-min inter-trial interval) for five days. For each trial, mice were placed in a black plastic start tube (7 cm diameter, 12 cm height) on the center of the maze. After 10 s, the start tube was raised and the buzzer was turned on to start the trial. Mice that were unable to locate the escape hole in 90 s were gently guided to it. Mice remained in the escape box for 10 s before being returned to their home cage.

### Tissue collection

For histological analyses, mice were anesthetized with an intraperitoneal injection of 150 mg/kg ketamine (Zoetis) and 20 mg/kg xylazine (Bayer) at 2, 7, or 14d after the second TBI, and brains were collected from perfused animals as described [46], except that 4% paraformaldehyde rather than neutral buffered formalin was used to post-fix hemisected brain tissue for histology. For biochemical analyses, brains were harvested as above at 6 h, 12 h, 2d, 7d, and 14d post-rTBI, longitudinally hemisected and rapidly frozen over dry ice and stored at -80°C until analysis.

### Microglial activation and silver staining

Silver staining and Iba-1 histology was performed as described [46]. Microglial morphology was quantified using fractal analysis [59] using ImageJ (NIH). Three to four microglia were randomly chosen from 40X-magnified Iba-1 stained images of olfactory nerve layer, corpus callosum, optic tract, and brachium of superior colliculus, converted to 8-bit images, and thresholded. Thresholded images were converted to outline images and analyzed using the FracLac plugin (Karperien, A., FracLac for ImageJ. http://rsb.info.nih.gov/ij/plugins/fraclac/FLHelp/Introduction.htm. 1999–2013.). The box counting method was used and the mean fractal dimension was analyzed. The number of microglia in each white matter regions was quantified by scanning the entire area using a Olympus BX61 microscope at 10X magnification with a ProScan motorized XY stage (Prior Scientific Inc, Rockland, MA). The component images were stitched together into a montage using ImagePro Plus image analysis software (Media Cybernetics Inc., Rockville, MD). The number of Iba1-positive cells were manually counted in the entire white matter track region of interest (ROI). The area of ROI was measured by ImageJ as pixels and scaled to mm^2^. Cell density was finally expressed as number of Iba-1 positive cells per mm^2^. Silver staining intensity was quantified using ImageJ (version 1.48, NIH) on 40X-magnified images of olfactory nerve layer, corpus callosum, and optic tract. The images were thresholded and the ROI was manually selected. The ratio of area of positive signal in ROI to total ROI area was reported as percent positive.

### Biochemical analyses

#### Tissue processing

For protein determination, half-brains were homogenized in RIPA lysis buffer as described [46].

#### Cytokine ELISA

Endogenous TNFα and IL-1β protein levels in the half-brain homogenates were quantified by commercial ELISA kits (BD Biosciences OptEIA 559603 and 555268, respectively) following the manufacturer’s instructions.

### Quantitative assessment of phosphorylated and total Tau by Simple Western analysis

Phosphorylated and total tau were assessed using an automated capillary electrophoresis-sized-based [60, 61] Simple Western system using the Wes machine (ProteinSimple, San Jose, CA). Simple Western is a gel-free, blot-free, capillary-based, automated protein immunodetection system that automates all the steps following sample preparation including sample loading, size-based protein separation, immunoprobing, washing, detection, and data analysis. All procedures were performed with manufacturer’s reagents according to the user manual. Briefly, 5 μL of RIPA lysate (2 μg of protein) was mixed with 1.2 μL of 5× fluorescent master mix and heated at 95°C for 5 min. The samples, blocking reagent, wash buffer, primary antibodies, secondary antibodies, and chemiluminescent substrate were dispensed into designated wells in the manufacturer-provided microplate. Following plate loading, separation and immunodetection were performed automatically using default settings. Data were analyzed with Compass software (ProteinSimple). Samples were immunodetected using following monoclonal antibodies (all kind gifts from Dr. Peter Davies, Albert Einstein College of Medicine, Manhasset, NY, USA): RZ3 is directed against tau protein phosphorylated at Thr231 (1:25), PHF1 is directed against tau protein phosphorylated at Ser396 and Ser404 (1:25), CP13 is directed against tau protein phosphorylated at Ser202 and Thr404 (1:25), and DA9 is directed against phosphorylation-independent (total) tau (1:5000). GAPDH (clone 6C5, 1:5000, Chemicon) was used as a loading control. Levels of phosphorylated and total tau were normalized to GAPDH. Levels of phosphorylated tau were expressed as fold difference compared to sham controls at the respective time points.

The rTBI protocol and post-rTBI end points are summarized in Additional file 6: Figure S3.

### Statistical analyses

The head kinematics data and graphs are presented as mean ± 95% CI. Behavioral data and graphs are presented as mean ± SEM. All other data and graphs are presented as mean ± SD unless otherwise specified. NSS, LRR, thigmotaxis, and rotarod data were analyzed using repeated measures two-way ANOVA followed by the Holm-Sidak post-hoc test, as animals were tested repeatedly until sacrifice. Passive avoidance and Barnes maze data were analyzed by repeated measures two-way ANOVA. Iba-1 and silver staining data were analyzed by two-way ANOVA followed by Tukey’s post-hoc test. For all the above statistical analyses, a *p* value of <0.05 was considered significant. Tau phosphorylation and cytokine protein expression at each post-rTBI time point was compared to the respective sham values by *t* test followed by Bonferroni correction of multiple comparison, with *p* value set to <0.01 (5 comparisons), for detecting statistical significance. Statistical analyses of behavioral data were performed using SigmaPlot (version 12.5, Systat Software Inc.). Statistical analyses for the rest of the data were performed using GraphPad Prism (version 6.04, GraphPad Software Inc).

## Electronic supplementary material

Additional file 1: Figure S1: CHIMERA allows unrestricted head motion during TBI. Before impact, the mouse head was freely supported on a foam pad in the supine position. Velcro straps were applied to the torso. Impact from the piston deflects the head, which then subsequently returns to its original position on the foam pad. The images were taken at 5,000 fps, at an angle perpendicular to the direction of impact and along the mouse sagittal plane. Each image shown was 12 ms apart. Head movement was tracked using two markers: a dental floss (red arrow in the first image) wrapped around the maxilla and a non-toxic paint (yellow arrow in the first image) applied at the lateral size of the head. (TIF 1 MB)

Additional file 2: Movie S4: Mouse head motion following impact using CHIMERA. The impact event was recorded at 5,000 fps. The video shows movement of the mouse head following impact by the pneumatically-driven piston. (ZIP 7 MB)

Additional file 3: Table S5: Comparison of kinematic parameters between rodent TBI models and human TBI [25, 31, 32, 57, 62–69]. (PDF 95 KB)

Additional file 4: Table S6: Neurological severity score tasks [29]. (PDF 157 KB)

Additional file 5: Figure S2: CHIMERA rTBI does not affect general mobility. General mobility was tested by the open field test at 1, 7 and 14d post-injury. No significant differences were observed between sham and rTBI mice in total distance travelled (A), number of lines crossed (B), or time spent immobile (C). Data are presented as the mean ± SEM and analyzed by repeated measures two-way ANOVA followed by Holm-Sidak post-hoc test. (TIFF 134 KB)

Additional file 6: Figure S3: Experimental Plan. The figure indicates the timeline for rTBI/sham procedure and behavioral, biochemical and histological end points at various post-rTBI time points used in this study. BM: Barnes maze, Iba-1: Iba-1 immunohistochemistry, NSS: neurological severity score, OF: open field behavior, PA: passive avoidance, RR: rotarod, SS: silver stain. (TIFF 176 KB)

## Abbreviations

CCI: Controlled-Cortical impact
CHI: Closed-Head injury
CHIMERA: Closed-Head Impact Model of Engineered Rotational Acceleration
DAI: Diffuse axonal injury
FP: Fluid percussion
LRR: Loss of righting reflex
mTBI: Mild traumatic brain injury
MVA: Motor vehicle accident
NFL: National football league
NSS: Neurological severity score
TBI: Traumatic brain injury
rTBI: Repeated traumatic brain injury.
