# Supplementary figures and images for: Merging pathology with biomechanics using CHIMERA (Closed-Head Impact Model of Engineered Rotational Acceleration): a novel, surgery-free model of traumatic brain injury
Source: Mol Neurodegener. 2014 Dec 1;9:55. doi: 10.1186/1750-1326-9-55 (PMC4269957; doi:10.1186/1750-1326-9-55)

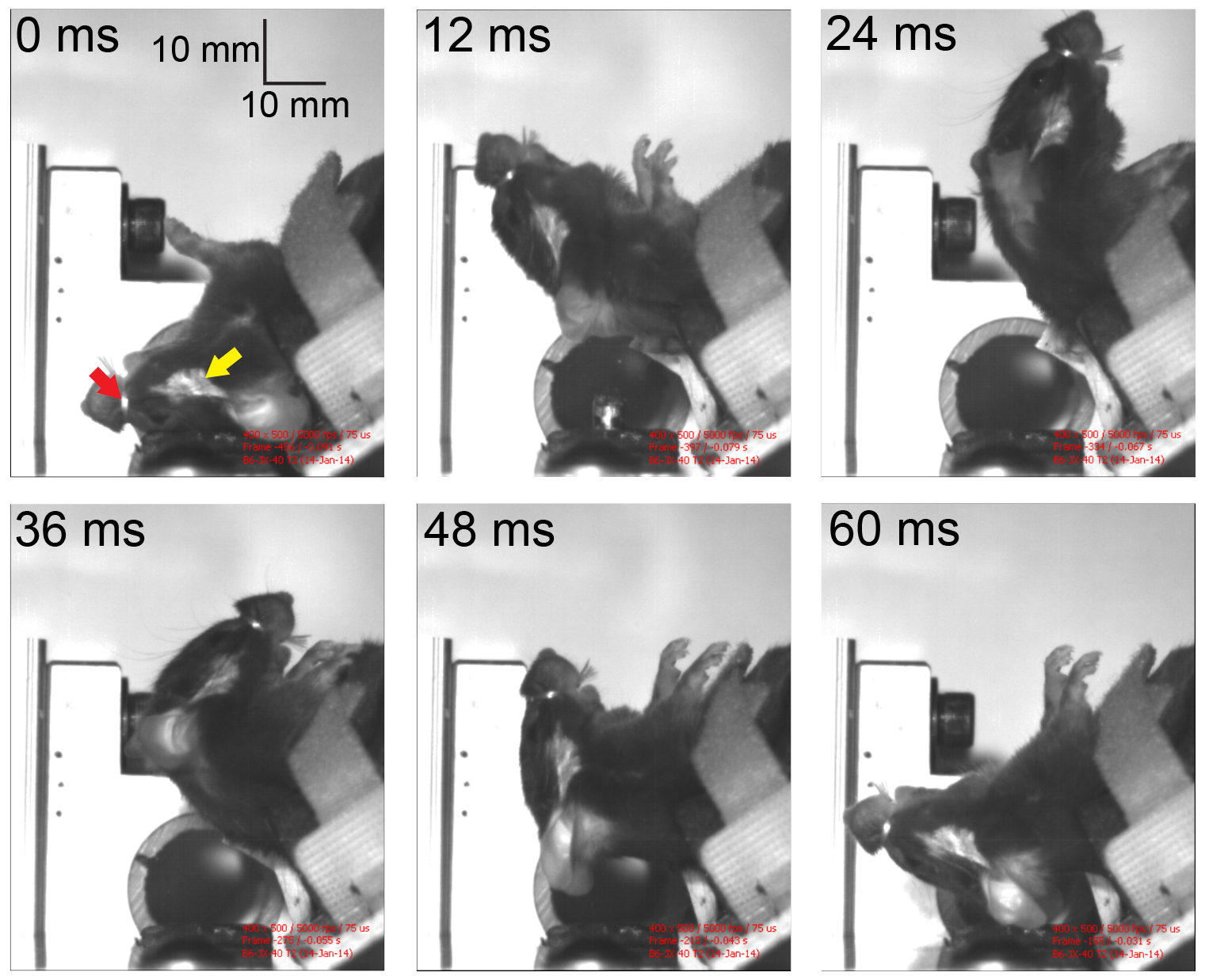

Supplement: Supplementary file 1 — Additional file 1: Figure S1: CHIMERA allows unrestricted head motion during TBI. Before impact, the mouse head was freely supported on a foam pad in the supine position. Velcro straps were applied to the torso. Impact from the piston deflects the head, which then subsequently returns to its original position on the foam pad. The images were taken at 5,000 fps, at an angle perpendicular to the direction of impact and along the mouse sagittal plane. Each image shown was 12 ms apart. Head movement was tracked using two markers: a dental floss (red arrow in the first image) wrapped around the maxilla and a non-toxic paint (yellow arrow in the first image) applied at the lateral size of the head. (TIF 1 MB) [file 13024_2014_563_MOESM1_ESM.tif]

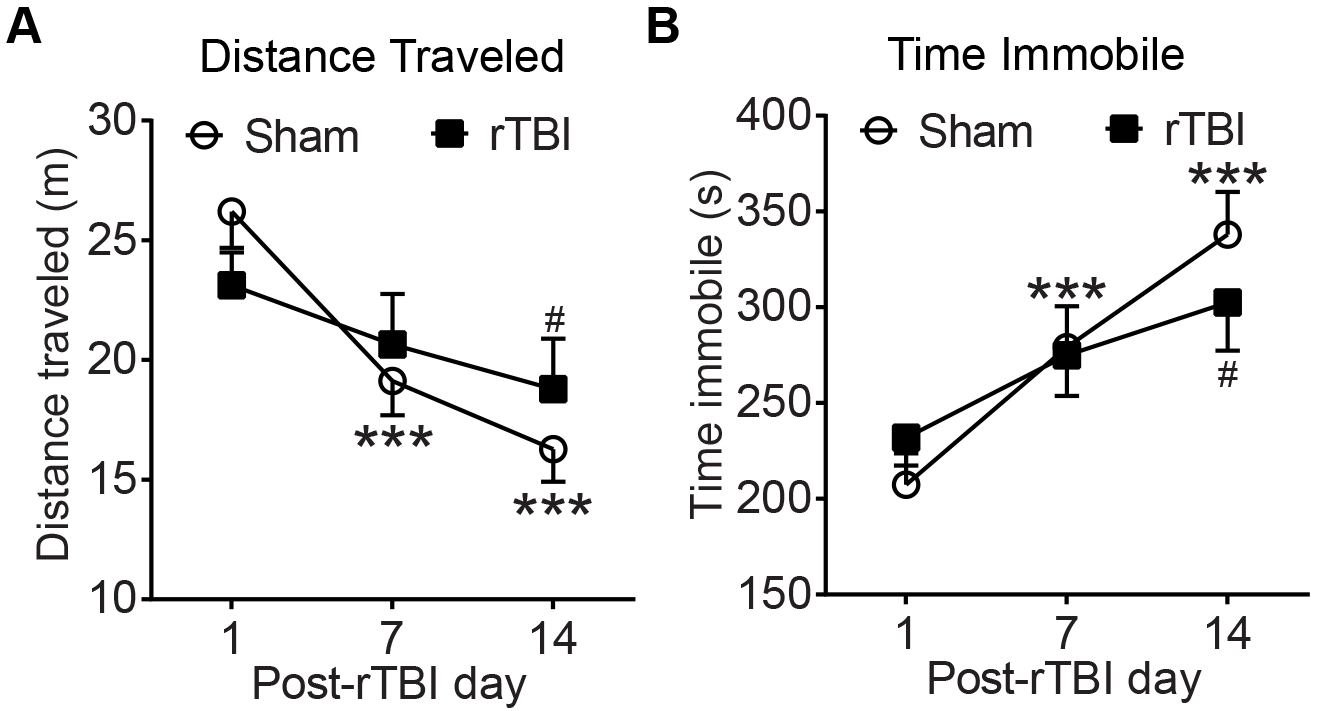

Supplement: Supplementary file 5 — Additional file 5: Figure S2: CHIMERA rTBI does not affect general mobility. General mobility was tested by the open field test at 1, 7 and 14d post-injury. No significant differences were observed between sham and rTBI mice in total distance travelled (A), number of lines crossed (B), or time spent immobile (C). Data are presented as the mean ± SEM and analyzed by repeated measures two-way ANOVA followed by Holm-Sidak post-hoc test. (TIFF 134 KB) [file 13024_2014_563_MOESM5_ESM.tiff]

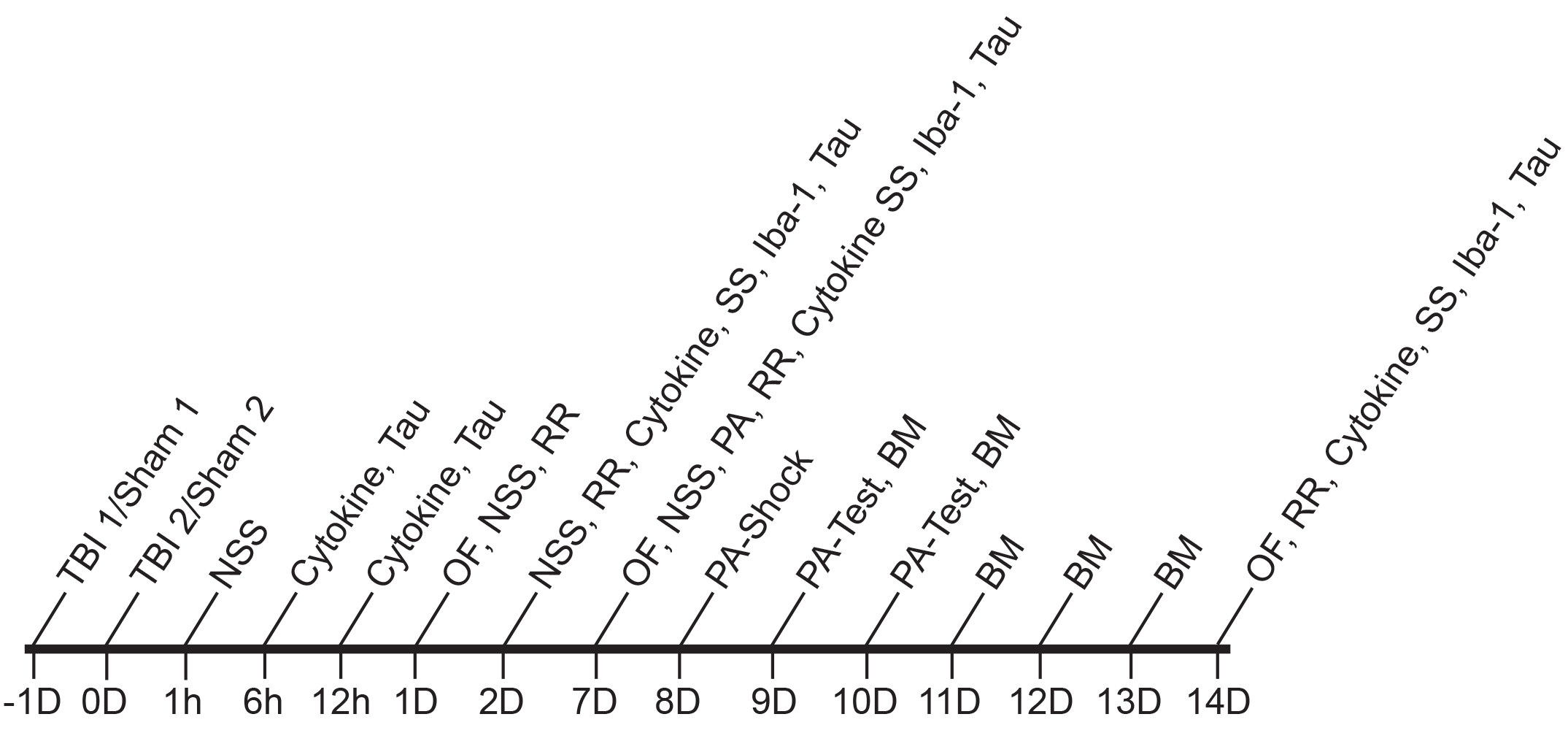

Supplement: Supplementary file 6 — Additional file 6: Figure S3: Experimental Plan. The figure indicates the timeline for rTBI/sham procedure and behavioral, biochemical and histological end points at various post-rTBI time points used in this study. BM: Barnes maze, Iba-1: Iba-1 immunohistochemistry, NSS: neurological severity score, OF: open field behavior, PA: passive avoidance, RR: rotarod, SS: silver stain. (TIFF 176 KB) [file 13024_2014_563_MOESM6_ESM.tiff]
