# Supplementary material for: Merging pathology with biomechanics using CHIMERA (Closed-Head Impact Model of Engineered Rotational Acceleration): a novel, surgery-free model of traumatic brain injury
Source: Mol Neurodegener. 2014 Dec 1;9:55. doi: 10.1186/1750-1326-9-55 (PMC4269957; doi:10.1186/1750-1326-9-55)
Supplement: Supplementary file 3 — Additional file 3: Table S5: Comparison of kinematic parameters between rodent TBI models and human TBI [25, 31, 32, 57, 62–69]. (PDF 95 KB) [file 13024_2014_563_MOESM3_ESM.pdf]

Additional file 3: Table S5. Comparison of kinematic parameters between rodent TBI models and human TBI.

Study	TBI Type	Species / Model	Head Kinematic Parameters (Scaled to Human)				HIC15
			ΔV (m/s)	Angular Velocity (rad/s)	Acceleration		
					Linear (*g*)	Angular (krad/s^2)	
**CHIMERA**	**Impact-acceleration**	**Mouse**	**6.6**	**22.6**	**27.9**	**1.4**	**40**
Goldstein et al (2012) [57]	Blast	Mouse	NA	36.2	NA	5.0	NA
Xiao-Sheng et al (2000) [62]	Non-impact rotation	Rat	NA	72.9	14.2	16.9	NA
Fijalkowski et al (2007) [63]	Non-impact rotation	Rat	NA	NA	NA	3.0	NA
Viano et al (2009) [25]	Impact-acceleration	Rat	NA	NA	93.5	NA	NA
Li et al (2010) [64]	Non-impact rotation	Rat	NA	NA	14.7	1.1	NA
Wang et al (2010) [65]	Non-impact rotation	Rat	NA	6.4	NA	1.1	NA
Davidsson et al (2011) [66]	Non-impact rotation	Rat	NA	NA	32.8-68.6	2.5-17.4	NA
Li et al (2011) [67]	Weight drop	Rat	NA	4.3-5.7	60.5-82.5	1.4-1.5	NA
Pellman et al (2003) [31, 32]	NFL concussion	Human	NA	34.8	97.8	6.4	250
Viano et al (2005) [68], Peng et al (2013) [69]	Olympic boxing – hook punch	Hybrid III dummy	3.1	29.3	71.2	9.3	79
	Olympic boxing – uppercut	Hybrid III dummy	2.8	17.5	24.1	3.2	17
	MVA involving pedestrian head impact (50% probability resulting in moderate injury)	Hybrid III dummy and FEM	NA	NA	116	11.4	825

NA: Data not available/not applicable

HIC15: Head Injury Criterion for 15 ms

ΔV: Change in velocity

MVA: Motor vehicle accidents

The table compares most commonly-reported head kinematic parameters between different TBI models in mice and rats. All the peak kinematic parameters are scaled to human values according to the equal velocity/equal stress approach. According to this approach, ΔV is unscaled, linear acceleration and angular velocity are scaled by 1 scale factor (λ), angular acceleration is scaled by λ^2. λ for mice and rats were 13.8 and 11.0, respectively. National Football League (NFL) concussion values and data from punches by Olympic boxers are included for comparison. Head Injury Criterion for 15 ms (HIC15) is included to compare the likelihood of head injury. HIC15 for the CHIMERA was calculated based on the definition:

$$\text{HIC} = \left\{ \left[\frac{1}{t_2 - t_1} \int_{t_1}^{t_2} a(t)dt \right]^{2.5} (t_2 - t_1) \right\}_{\max}$$

where linear acceleration (*a*) is in *g* and scaled by λ ,time (*t*) is in seconds and scaled by 1/λ, and t_1 and t_2 are determined to give maximum value to the HIC function such that t_2 - t_1 = 15 ms.
