# Supplementary material for: Merging pathology with biomechanics using CHIMERA (Closed-Head Impact Model of Engineered Rotational Acceleration): a novel, surgery-free model of traumatic brain injury
Source: Mol Neurodegener. 2014 Dec 1;9:55. doi: 10.1186/1750-1326-9-55 (PMC4269957; doi:10.1186/1750-1326-9-55)
Supplement: Supplementary file 4 — Additional file 4: Table S6: Neurological severity score tasks [29]. (PDF 157 KB) [file 13024_2014_563_MOESM4_ESM.pdf]

**Additional file 4: Table S6. Neurological severity score tasks [29]**

| Task | Description | Points (success/failure ) |
|---|---|---|
| Exit circle | Ability and initiative to exit a circle of 30 cm diameter within 3 min | 0/1 |
| Monoparesis/hemiparesis | Paresis of upper and/or lower limb of the contralateral side | 0/1 |
| Straight walk | Alertness, initiative and motor ability to walk straight | 0/1 |
| Startle reflex | Innate reflex; the mouse will bounce in response to a loud hand clap | 0/1 |
| Seeking behavior | Physiological behavior as a sign of 'interest' in the environment | 0/1 |
| Beam balancing | Ability to balance on a beam of 7 mm width for at least 10 s | 0/1 |
| Round stick balancing | Ability to balance on a round stick of 5 mm diameter for at least 10 s | 0/1 |
| Beam walk: 3 cm | Ability to cross a 30-cm long beam of 3 cm width | 0/1 |
| Beam walk: 2 cm | Same task, increased difficulty on a 2-cm wide beam | 0/1 |
| Beam walk: 1 cm | Same task, increased difficulty on a 1-cm wide beam | 0/1 |
| Maximal Score | | 10 |
